# The Innate Immune-Related Genes in Catfish

**DOI:** 10.3390/ijms131114172

**Published:** 2012-11-02

**Authors:** Lei Gao, Chongbo He, Xueguang Liu, Hao Su, Xianggang Gao, Yunfeng Li, Weidong Liu

**Affiliations:** 1 Liaoning Ocean and Fisheries Science Research Institute, Dalian 116023, China; E-Mails: seamas_gao@yahoo.com.cn (L.G.); shuhao136@yeah.net (H.S.); xiangganggao@hotmail.com (X.G.); yunfengli@126.com (Y.L.); cnliu51@tom.com (W.L.); 2 Key Laboratory of Marine Fishery Molecular Biology of Liaoning Province, Dalian 116023, China; 3 Liaoning Fisheries Technology Extension Station, Shenyang 110031, China; E-Mail: lxueguang@163.com

**Keywords:** catfish, channel catfish (*Ictalurus punctatus*), innate immunity, antimicrobial peptide, complement, lectin, cytokine, RNA-seq, gene expression profiling

## Abstract

Catfish is one of the most important aquaculture species in America (as well as in Asia and Africa). In recent years, the production of catfish has suffered massive financial losses due to pathogen spread and breakouts. Innate immunity plays a crucial role in increasing resistance to pathogenic organisms and has generated increasing interest in the past few years. This review summarizes the current understanding of innate immune-related genes in catfish, including pattern recognition receptors, antimicrobial peptides, complements, lectins, cytokines, transferrin and gene expression profiling using microarrays and next generation sequencing technologies. This review will benefit the understanding of innate immune system in catfish and further efforts in studying the innate immune-related genes in fish.

## 1. Introduction

Catfish (Order Siluriformes) is a diverse group of ray-finned fish representing more than 3000 species, 478 genera and 36 families [1]. It is one of the most important aquaculture species worldwide. Several catfish species are used in aquaculture including channel catfish (*Ictalurus punctatus*) [2], blue catfish (*Ictalurus furcatus*) [3], walking catfish (*Clarias fuscus*) [4] and striped catfish (*Pangasianodon hypophthalmus*) [5]. The culture of catfish accounted for approximately 3,201,172 tons of the production and 4,892,359,000 dollars of the profit, respectively [6]. Nevertheless, catfish production suffered massive financial losses due to pathogen spread and breakouts. For instance, enteric septicemia of catfish (ESC), caused by a Gram-negative intracellular bacterium *Edwardsiella ictaluri*, resulted in 40–50 million dollars of annual losses in profits to catfish producers [7,8]. To clarify the mechanisms of resistance to *E. ictaluri*, many different studies have been conducted in striped catfish [9–13]. Immunity research in catfish is of great interest in recent years and contributes to an advance in catfish breeding.

The innate immune system is the only defense weapon in invertebrates and plays an instructive role in the acquired immune system of higher vertebrates [14]. Teleost fish serve a key role as the bridge between innate and adaptive immune responses in that they are the earliest class of vertebrates possessing the elements of both innate and adaptive immunity [15]. In teleost, innate immunity occupies a more important position for the initial protection against pathogen invasion, due to the constraint on adaptive immunity in suboptimal environments [16]. The studies on relevant genes of innate immunity have been conducted in many commercial fish species, such as rainbow trout (*Oncorhynchus mykiss*) [17–19], puffer fish (*Tetraodon nigroviridis*) [20], large yellow croaker (*Pseudosciaena crocea*) [21,22], and channel catfish [16,23,24].

Catfish plays an important role in fish immunology research and many advances have been achieved in genomic study [25,26]. One example is the case of channel catfish, a model species for the study of comparative immunology [27,28]. Moreover, catfish remains to be the only fish species wherein clonal functionally of distinct lymphocyte cell lines have been established [29–31]. In catfish, innate immunity has built a set of complete defense system, proving beneficial in increasing resistance to pathogenic organisms [32], such as *E. ictaluri* and *Flavobacterium columnare*[33]. More recently, a large number of immune-relevant genes for innate immunity have been characterized in catfish, such as those encoding pattern recognition receptors, antimicrobial peptide, complements, lectins and cytokines [34–38] (Table 1). They provide immediate defense against infection and constitute an evolutionarily older defense strategy. Individual variations of these genes were observed in numerous studies, resulting in different activities of the gene productions. These variations in different individuals may account for the differences in resistance or susceptibility to disease, which is responsible for the health condition of catfish when suffered pathogenic organisms. Hence, these traits could be used to select disease resistance in breeding programs, and are important for the health management [15,39]. This review will focus on the current advances of innate immune-related genes of catfish and the future efforts.

## 2. Current Advances of Innate Immune-Related Genes in Catfish

### 2.1. Pattern Recognition Receptors

Pattern recognition receptors (PRRs) are responsible for recognizing microbial pathogens according to sensing structures conserved among microbial species, known as pathogen-associated molecular patterns (PAMPs), including lipopolysaccharide (LPS) or peptidoglycan (PGN) in bacterial cell walls, β-1,3-glucan on fungal cell walls and double-stranded RNA from viruses [63,64]. Recent studies showed that PRRs contribute to recognition of endogenous molecules released from damaged cells, named damage associated molecular patterns (DAMPs), such as oxidized phospholipids and oxidized cholesteryl esters [65]. So far, several different classes of PRRs have been identified, including transmembrane proteins such as the Toll-like receptors (TLRs) and C-type lectin receptors (CLRs), as well as cytoplasmic proteins such as the Retinoic acid-inducible gene (RIG)-I-like receptors (RLRs) and NOD-like receptors (NLRs). To date, at least 17 different TLRs have been identified in teleosts; zebrafish (*Danio rerio*) [66,67], rainbow trout [68,69], common carp (*Cyprinus carpio*) [70], pufferfish (*Takifugu rubripes*) [71], channel catfish [40–42] and Atlantic salmon (*Salmo salar*) [72]. Moreover, teleost fish were found to possess an additional group of NLRs and the foundational framework for analysis was provided [73,74].

#### 2.1.1. Toll-Like Receptors

TLRs are the first PRRs to be identified and are evolutionarily conserved [75,76]. To date, a total of 13 (TLR1 to TLR 13) and five (TLR2, TLR3, TLR5, TLR20 and TLR21) functional TLRs have been characterized in mammals and catfish, respectively [24]. The first study on TLRs in catfish was carried out by Bilodeau and Waldbieser [40], who identified TLR3 and TLR5 from the channel catfish cDNA fry library and conducted expression analysis upon challenge with *E. ictaluri*. Despite no direct relationship between susceptibility to ESC and this two TLRs expression observed, it was suggested that TLR3 was associated with a more widespread immune function and TLR5 contributed to the aggregation of macrophage during *E. ictaluri* infection [40,77]. TLR3 and TLR 5 were suggested to play a role in host response to bacterial challenges in hybrid catfish, as well as during embryogenesis and early development of hybrid and channel catfish [78,79]. TLR3 was found to be induced and expressed highly in stomach, the primary uptake point of *E. ictaluri*, which demonstrated the more important role TLR3 played in innate immunity than previously thought [79]. Some other TLRs’ cDNA and gene have also been cloned, sequenced and characterized in catfish, including TLR2 [41], TLR5S (the soluble isoform of TLR5), TLR20, TLR21 [42], and adaptor protein of TLR3 [80]. TLR2, generally recognizing lipopeptides on the surface of most Gram-positive bacteria, was suggested to be involved in the response, after infection, with a Gram-negative bacterium (*E. ictaluri*), while the mechanisms of this response were not clear yet. After that, Pridgeon *et al.*[24] determined the expression pattern of all five TLRs under acutely infected conditions and demonstrated the important roles TLRs play in response to acute infection. It is not quite conserved for the genomic organizations of TLRs genes between catfish and other species. Taking TLR2 for example, it does not contain any introns in catfish, but has one, two and ten introns in human, murine and pufferfish [20], respectively.

#### 2.1.2. NOD-Like Receptors

NLRs are a recently identified large group of intracellular PRR family in vertebrates. There are three subfamilies detected in NLRs: the NODs (nucleotide-binding oligomerization domain) and IPAF (ICE protease-activating factor) (CARD), the NAIPs (neuronal apoptosis inhibitory proteins) (BIR), and the NALPs (NACHT domain-, leucine-rich repeat-, and PYD-containing proteins) (PYD) [81,82]. In fish, NLRs were first identified from zebrafish, including NLR-A, NLR-B and NLR-C, and NLR-C appeared to be unique in teleost fish [73]. There is little known about NLRs in catfish until recently. Sha *et al.*[43] reported the characterization of 5 NLRs in channel catfish, conducting analysis on structure, phylogeny and expression. Among NOD1, NOD2, NLRC3, NLRC5 and NLRX1, NOD 1 appeared to be induced after injection with *E. ictaluri*, catalogued as a component in the response to intracellular pathogen injection [43]. Then, a more extensive study was carried out, which identified 22 NLRs involved in several different subfamilies [37]. The similar expression pattern of 22NLRs after infection suggested the co-regulation of these genes [37].

#### 2.1.3. Retinoic Acid-Inducible Gene (RIG)-I-Like Receptors

RLRs are important PRRs that detect nucleotide PAMPs of invading viruses [83]. Unlike TLRs detecting the RNA species present within endosome, the RLRs recognize viral RNA in the cytoplasm of both immune and non-immune cells [84]. The RLR family contains three coding genes: retinoic acid-inducible gene I (RIG-I), melanoma differentiation associated antigen 5 (MDA5), and laboratory of genetics and physiology 2 (LGP2) [85]. In recent years, RLRs have been reported in many teleost species [86–90], as well as in catfish. Rajendran *et al.*[38] identified three RLRs in channel catfish, including RIG-I, MDA5 and LGP2. These authors confirmed the presence of significant increases in expression of RLRs after bacterial infection, not just after viral infection, which indicated their roles in both antiviral and anti-bacterial immune responses.

### 2.2. Antimicrobial Peptides

Antimicrobial peptides (AMPs) are an evolutionarily conserved component of the innate immune system, widespread in all classes of life as defense mechanisms [91]. They demonstrate a wide range of activity against a number of pathogenic organisms, while with little or no toxicity to host cells [92]. The AMPs found in human are mainly defensins and cathelicidins. Human defensins are divided into two families, including α-defensins, distributed in neutrophils and intestinal Paneth cells, and β-defensins, generated by the epithelia of several organs [93]. To date, there are five different classes of AMPs detected, according to the structural features. Roughly 1200 AMPs have been characterized from various organisms, and the numbers of that identified in teleost fish increased rapidly in recent years [94–99], especially in catfish.

The cysteine-rich AMPs play an important role in the host innate immune response against microbial invasion and have been extensively studied from various organisms. Hepcidin [44] and liver-expressed antimicrobial peptide 2 (LEAP-2) [45], members of cysteine-rich AMPs family, were sequenced and characterized from both channel catfish and blue catfish (*Ictalurus furcatus*). In channel catfish, the amino acid sequences and gene organization of both hepcidin and LEAP-2 were conserved between catfish and other organisms, and the expressions of both two cysteine-rich AMPs were induced in a tissue-specific manner after infection with *E. ictaluri*[44,45]. The channel catfish hepcidin gene was expressed in a wide range of tissues, unlike those in some fish and mammals, which are predominantly expressed in liver [97,100]. Its expression was also detected early during embryonic and larval development, suggesting its possible role as self-generated AMPs in protecting embryos against bacterial pathogens. Three distinct NK-lysin transcripts, as the fifth class of AMPs and only one copy existing in human, was identified from catfish by Wang *et al.*[23,46], with the encoding genes identified, sequenced, and characterized. The expressions of three catfish NK-lysin genes, tripled in tandem, were tissue specific and different, suggesting the spatial partitioning from each other [23,46]. In addition, bactericidal permeability-increasing protein (BPI) [47] and parasin I [101,102] also have been characterized from catfish. It was demonstrated that BPI had the function in beraking the rapid inflammatory responses elicited by ESC infection, according to its upregulated expression pattern [47]. After that, Pridgeon *et al.*[36] determined the relative transcriptional levels of all seven AMPs genes above in response to *E. ictaluri* infection, suggesting the important role of hepcidin in the channel catfish defense against *E. ictaluri* infection.

Histone-like proteins (HLPs), one of the most prevalent AMPPs in fish, have been characterized in catfish including histone-H2A-like (parasin I) [103] and histone-H2B-like [101] protein, which have recently been definitively identified as histones [104]. HLP-1 upregulation was considered as a promising tool in aquaculture for enhancing the resistance of fish to disease [105]. Moreover, hemoglobin-derived AMPs [16] and pelteobagrin [91] have been identified in channel catfish and yellow catfish (*Pelteobagrus fulvidraco*), respectively. Hemoglobin-derived AMPs was considered to play a significant role in innate immune response, while the expression analysis of the pelteobagrin is needed in the future work.

Despite the progress in AMPs in catfish, little is known about some other groups of AMPs in fish, such as defensins and natural resistance-associated macrophage protein (Nramp). Defensins have been identified in common carp [106], rainbow trout [17,19], zebrafish [107], olive flounder (*Paralichthys olivaceus*) [108], medaka (*Oryzias latipes*) [109] and orange-spotted grouper (*Epinephelus coioides*) [110]. Likewise, Nramp genes have been discovered in common carp [111], turbot (*Scophthalmus maximus*) [112] and Japanese flounder (*Paralychthys olivaceus*) [113]. Further studies are necessary to identify these genes in catfish.

### 2.3. Complement

Complement occupies a key position in the innate immune system, consisting of numerous soluble membrane-bound proteins. Its functions are very extensive, including microbial killing, phagocytosis, inflammatory reactions, immune complex clearance, and antibody production [114]. Three different ways are found to activate the complement system: the classical pathway, the mannan-binding lectin pathway, and the alternative pathway. The classical pathway is mediated by antibodies. The mannan-binding lectin pathway is activated by host lectins according to recognition and binding of microbial carbohydrate motifs. In contrast, the alternative pathway is activated by numerous foreign molecules and is continuously active [48,115]. Despite the fact that the complement system has been studied extensively in mammals, its role in immune system of lower vertebrates remains largely unknown [115]. The studies of complement in catfish are increasing in recent years, focused mainly on channel catfish.

It has been proved that teleost fish possess fully developed complement activation pathways. The first subcomponent in classical pathway, C1q, was first identified in channel catfish [116]. The subsequent research of detailed structure and function of Cq1 were mainly focused on zebrafish [117] and mandarin fish (*Siniperca chuatsi*) [118]. The alternative complement pathway of channel catfish was well-studied in the early 1990s [119]. The effect of an alternative complement pathway on the case, such as neutrophil and monocyte phagocytes from peripheral blood [120], and permeability of cytoplasmic membranes in *Escherichia coli*[121], has been determined. In addition, the activity of alternative complement pathway in catfish was significantly affected by bacterial sialic acid [122] and *Salmonella paratyphi*[123]. Complement components C3 and C4, the central protein of complement system, have been isolated and characterized in channel catfish [124]. It is the first time to present structural evidence for C4 in the bony (channel catfish) and cartilaginous fish (nurse shark, *Ginglymostoma cirratum*), and demonstrate the difference between C3 and C4 before the divergence of bony and cartilaginous fish [124]. The initiating component for activating mannan-binding lectin pathway, mannose-binding lectin, has been characterized in channel catfish [35], but still not forceful to confirm the existence of the mannan-binding lectin pathway.

In spite of the effective role of the complement system in response to foreign pathogens, self-destruction sometimes occurs resulting in immunological diseases [125,126]. A whole array of specific inhibitors and other factors were identified from various organisms, regulating the activity of complement system. Complement factor I [48] and complement membrane attack complex inhibitor CD59 [49], both as inhibitors in complement system, were sequenced and characterized in channel catfish. Complement factor I and CD59 were showed to be expressed constitutively in various tissues and organs, presenting different conformance from that in mammals. These results are consistent with those discovered in large yellow croaker [127,128] and rainbow trout [129]. In addition, three factors in alternative complement pathway of channel catfish, Bf/C2A, Bf/C2B and Df, were identified and characterized, the expression patterns of which demonstrated the key role in immune response to bacterial infection [34]. C3 and Bf were regarded as maternal factors in several fish species, including spotted wolfish (*Anarhichas minor*) [130], rainbow trout [131], common carp [132], grass carp (*Ctenopharyngodon idellus*) [133], Atlantic salmon [134] and zebrafish [135,136], which were not confirmed in catfish yet.

### 2.4. Lectin

In host defense, the recognition of pathogen-specific carbohydrate chains mainly relies on the activity of lectins, which play an important role in innate immunity and disease resistance [137–139]. The carbohydrate molecules on the pathogens surface is combined with lectins and removed through phagocytosis and oxidative burst activities [64,140,141]. Lectins are divided into six families based on the carbohydrate recognition domain: legume lectins, cereal lectins, P-, S-, C-type lectins and pentraxins [142,143]. In addition, lectins could activate a complement system through the so called lectin pathway, by forming complexes with mannose-binding lectin-associated serine proteases in plasma [144]. In fish, lectins such as C-type lectins, galectins, F-type lectins, rhamnose-binding lectins, and intelectins have been characterized [145], mainly derived from serum, plasma, skin mucus, egg surfaces and egg components [146–149].

In channel catfish, C-type and L-type lectins have been well-studied in recent years. Mannose-binding lectin (MBL), an acute-phase protein produced by liver hepatocytes, was purified by affinity chromatography, characterized and quantified [35,150]. Human MBL is assembled from a single polypeptide chain, consisting of a short *N*-terminal cysteine-rich region, a collagenous region, a 34-residue hydrophobic stretch, and a *C*-terminal C-type lectin domain [151]. Based on the difference in levels of serum MBL between channel catfish and blue catfish challenged with *E. ictaluri*, it is suggested that MBL could be used as a genetic marker for selection of disease resistance in the different strains of catfish used in aquaculture [152]. Meanwhile, three L-type lectins, ERGIC-53, VIP36 and VIP36-like, were cloned and characterized in channel catfish, of which the expression patterns of significant up-regulation upon infection with *E. ictaluri* indicated the involvements in the immune responses after infection with *E. ictaluri*. According to phylogenetic analysis, higher similarity (55%–65%) was observed between the protein sequences of teleost LMAN2-like (VIP36-like) a genes and LMAN2-like b genes, suggesting a duplication event in most fish [153]. In walking catfish, the calcium dependent lectins were isolated from the serum and characterized, including galactose binding serum lectin [154] and CBL (*C. batrachus* lectin) [155]. As a member of lectins, microfibrillar-associated protein 4 (MFAP4), was isolated and characterized in channel catfish, and the novel role for MFAP4 in immune responses was detected [156].

Intelectin (IntL), a secreted soluble glycoprotein belonging to the lectin family, is a recently identified member of the galectins [157]. Takano *et al.*[50] characterized two intelectin genes in both channel catfish and blue catfish, IntL1 and IntL2, exhibiting highly differential patterns of tissue expression and regulation after infection with *E. ictaluri*. A skin mucus lectin was also found in Japanese catfish *Silurus asotus*, exhibiting calcium-dependent mannose-binding activity and functioned in self-defence against bacteria in the skin surface [51]. In addition, some lectins associated with egg, mainly focused on catfish (*S. asotus*) egg lectin (SAL), were also isolated and characterized in catfish [158–162].

### 2.5. Cytokines

#### 2.5.1. Chemokines

Chemokines, the superfamily of chemotactic cytokine, are a family of small proteins produced by infected tissues in the early stages of infection [136]. They play a key role in recruiting immune effector cells to the focus of infection and injury [163], and the role as a bridge between innate and adaptive responses. Chemokines are defined by the presence of four conserved cysteine residues, which exist in majority of the structures [164]. According to the arrangement of the first two of these conserved cysteine residues, chemokines are divided into four subfamilies: CXC, CC, C and CX3C. Chemokines were also found to be necessary to translate an innate immune response into an acquired adaptive response in that the chemokine system appeared about 650 million years ago, at the emergence of vertebrates [165]. There are roughly 50 chemokines and 20 receptors identified in humans [166], and the research in teleost increased rapidly in recent years [167,168]. According to phylogenetic analysis, seven large groups of fish CC chemokines have been established: the CCL19/21/25 group, the CCL20 group, the CCL27/28 group, the CCL17/22 group, the macrophage inflammatory protein (MIP) group, the monocyte chemotactic protein (MCP) group and a fish-specific group [168,169].

Among the four subfamilies of chemokines, CXC and CC chemokines have been better and deeply studied. A CXCL10-like chemokine is the first one discovered in catfish [52]. Although channel catfish were much more susceptible to *E. ictaluri* than blue catfish, the CXCL10-like chemokine was induced strongly in channel catfish compared to blue catfish, suggesting that the CXCL10-like chemokine did not contribute to the resistance against *E. ictaluri*[52]. A catfish gene resembling interleukin-8 (IL-8), the first CXC chemokines to be discovered in fish [170], was identified in both channel catfish and blue catfish [53]. The expression of IL-8 was dramatically induced after challenged with the bacterial pathogen *E. ictaluri*. Another three CXC chemokines, CXCL12, CXCL14 and CXCL2-like chemokine, were characterized by Baoprasertkul *et al*. [54]. The first two chemokines were clearly orthologous, whereas a clear orthology could not yet be established for the third one. Concerning CC chemokines, the first 14 of them were identified in both channel catfish and blue catfish by analysis of ESTs [55]. Subsequently, a further study characterized 12 more distinct CC chemokine genes, bringing the total to 26, two more than known from humans [57]. All 26 CC chemokines were mapped to bacterial artificial chromosome (BAC) clones, suggesting the extensive duplication at various levels rather than only highly clustered in the catfish genome [171]. The study of complete genomic sequences and structures was conducted for 23 CC chemokine genes, indicating the different expression patterns in tissues [56]. In addition, the first chemokine receptors in catfish, CXCR4, has been identified more recently [58]. In that five CXCL chemokines of catfish have been characterized, the findings of CXCR4 would further explore the signaling pathways of chemokine.

#### 2.5.2. Interferons

Interferons (IFNs) are secreted proteins and crucial elements of the immune system sharing antiviral properties, as well as the ability of tumor suppression and immune modulation [172,173]. Based on the relevant cognate receptors and initiating immune responses, IFNs are divided into three distinct types, including IFNs of type I, II and III. There are numerous differences among them in genomic structure and the cell sources producing them [174]. For example, unlike type I IFN produced widely as a major role in the first line of defense against virus infection [175], type II IFN is produced by NK cells during innate responses and by T cells during the adaptive immune response [176]. In contrast to type II IFNs mediated cell immunity, type I and III IFNs mainly initiate the innate response against viral infection according to triggering specific signaling pathways [174]. In recent years, considerable advances have been achieved in the study of the fish IFN system, especially the roles of type I and II IFN in response to viral infection.

In catfish, four genes were observed to encode the virus-induced IFNs [59], and the numbers in zebrafish [177] and Atlantic salmon [178] were 4 and 11, respectively. The first IFN discovered in catfish was designated CF IFN-1, identified from a catfish EST library [179]. According to the infection of channel catfish ovary (CCO) cells with UV-inactivated catfish reovirus or exposure to double stranded RNA, the upregulation of CF IFN mRNA and appearance of antiviral factor were observed. The authors speculated that the identified catfish cDNA was an IFN homolog [179]. Subsequently it was found that, a signal sequence within the catfish homolog was lacked in CF IFN-1, suggesting that CF IFN-1 encoded a non-secreted protein and might represent an expressed pseudogene [59]. Based on Southern blot analysis, Long *et al.*[59] identified three novel cDNAs encoding CF IFN-2, -3, and -4. In contrast with CF IFN-3, without a signal sequence just like CF IFN-1, CF IFN-2 and IFN-4, it appeared to encode full-length, signal sequence-bearing functional genes. After that, two distinct type II IFN, IFN-γ1 and IFN-γ2, were identified in catfish [60]. The authors presented the perspective that it was the first time to demonstrate NK and T cells synthesizing IFN-γin any lower vertebrate species, consistent with that in mammalian systems [60].

#### 2.5.3. Interleukin

Interleukins are a large group of cytokines including both secreted proteins and signaling molecules, which are first seen to be expressed by leukocytes. Interleukin-1 (IL-1) serves a key role in early inflammatory response and induces a number of reactions leading to inflammation [136]. In mammals, IL-1 family is comprised of 10 ligand proteins and 10 receptor molecules [180,181], three members of which, IL-1α, IL-1β and the IL-1 receptor antagonist (IL-1ra), have been deeply studied in the past [15]. IL-1β is synthesized primarily in monocytes, as well as many other cell types, and has been identified in about 13 teleost species [182]. As characterized in several fish species, such as rainbow trout [183], common carp [184], sea bass (*Dicentrarchus labrax*) [185,186] and yellowfin sea bream (*Acanthopagrus latus*) [187], only one IL-1β gene and a variant appeared to exist in fish. However, in channel catfish, two distinct cDNAs encoding catfish IL-1β were identified, the encoding genes of which were identified, sequenced and characterized [61]. Both genes were duplicated in a tandem fashion and widely expressed, but presented different expression patterns [61]. In addition, a catfish gene of interleukin-8, at the same time that of CXC chemokines, was identified and characterized from both channel catfish and blue catfish [53].

#### 2.5.4. Tumor Necrosis Factor

The tumor necrosis factors (TNFs) are cytokines involved in innate immunity, causing cell apoptosis. TNFα, the best known member of TNFs, is a type II transmembrane glycoprotein binding to two receptors, TNFR-1 and TNFR-2. It appears to be the key regulator and effector in immune responses by regulating cell death and survival [188]. TNFα has been identified in several fish species, including mandarin fish [189], zebrafish [190], common carp [191] and turbot [136,192]. A TNFα-like gene was identified in channel catfish, which encoded a propeptide of 230 amino acids and a mature peptide of 162 amino acids [193]. This research supported the perspective that TNFα and β genes separated after the divergence of mammals and teleosts, in that phylogenetic analysis placed teleost TNF sequences within their own cluster apart from mammalian TNFα and β genes. TNFα mRNA in catfish was found to be expressed in all tissues tested in the study, in the manner similar to that of mammals [193]. Moreover, expression analysis of TNF response to *E. ictaluri* infection was characterized in channel catfish families with high and low susceptibility [194]. It was found that the expression of TNF increased significantly at 48 h post-challenge in both high and low susceptibility families, and decreased by 72 h [194].

### 2.6. Transferrin

Iron plays a crucial role in a wide range of metabolic processes in host organisms, as well as in pathogenic organisms. However, the concentration of extracellular free iron remains at considerably low levels to restrict the assimilation by pathogen, in that the blood protein transferrin has high affinity for iron and transports iron to tissues as required [195–197]. Transferrin is widespread in the serum and secretions of all vertebrates with high degree of genetic polymorphism [198]. It is responsible for iron metabolism and level maintaining, and transporting iron to tissues as required [62]. Although iron bound to transferrin is only about 4 mg in human body, transferrin is one of the most important iron pools with high rate of turnover. In channel catfish, the transferrin gene was identified, sequenced and characterized [62]. This transferrin expression was significantly up-regulated after infection with *E. ictaluri*, as well as with co-injection of iron-dextran and *E. ictaluri*[62]. The cDNA of transferrin in Gunther’s walking catfish (*Clarias macrocephalus*) was also cloned and characterized [199]. The transferrin expression was only detected in the liver of both male and female catfish [199]. Moreover, ferritin H gene was completely sequenced and characterized in channel catfish [200]. Genetic organization of ferritin H shares high similarity with mammalian and zebrafish genes, and it has an important role in iron metabolism and immunity.

## 3. Gene Expression Profiling

Gene expression profiling, through the dual approaches of transcriptomics (RNA profiling) and proteomics (protein profiling), has played an essential role in understanding the complex biological processes [201]. It has provided us with insight into the different roles of various genes in immune responses and other metabolic activities. The transcriptome, representing a key link between information encoded in DNA and phenotype, has been more intensively studied in catfish and other fish species. The traditional methods for transcriptome analysis are relying on hybridization to capture transcripts of interest, and used the tools such as Northern blots, reverse-transcription PCR (RT-PCR), expressed sequence tags (ESTs), and serial analysis of gene expression (SAGE), which were of low efficiency.

Microarrays first make the large-scale analysis of the transcriptome possible and have produced much more important information about transcriptome deployment and gene expression [202–206]. The application of microarrays has been used in many fish species, including zebrafish [207,208], turbot [209,210], rainbow trout [211], and Japanese medaka [212]. In catfish, utilizing a new high-density *in situ* oligonucleotide microarray (28K), the acute phase response (APR) in liver following infection with *E. ictaluri* was evaluated [213]. The analysis of microarray results revealed a well-developed APR in catfish, including intelectin, hemopexin, haptoglobin, ferritin, and transferrin, with particularly high upregulation (>50-fold) of genes involved in iron homeostasis. The majority of complement cascade, PRRs and chemokines were observed to be expressed differently following infection. Subsequently, the microarray analysis (28 K) of gene expression in channel catfish and blue catfish has been conducted [3]. A whole array of multifaceted responses to infection could be observed, including encompassing the complement cascade, iron regulation, inflammatory cell signaling, and antigen processing and presentation. For the first time, it reported the induction of several components of the MHC class I-related pathway following infection with an intracellular bacterium. Pridgeon *et al.*[214,215] conducted the global analysis of gene expression and transcription in vaccinated channel catfish, which suggested the important roles of differentially regulated genes in the protection of channel catfish against *E. ictaluri*.

More recently, however, researchers are increasingly turning to next generation sequencing technologies, such as Transcriptome Sequencing (RNA-seq), using the Illumina Genome Analyzer platform, ABI Solid Sequencing or Life Science’s 454 Sequencing, which has considerable advantages for examining transcriptome structure. It utilizes the throughput capacity of next generation sequencing to sequence transcripts and quantify expression levels [216]. The RNA-seq sequencing prices are declining rapidly, and it may change the way in identification of immune-related genes and signaling networks. Related studies have been performed in zebrafish [217,218], common carp [219], sea bass [220] and rainbow trout [221]. In catfish, the role of the intestinal epithelial barrier following *E. ictaluri* challenge was characterized using high-throughput RNA-seq, which obtained 2719 genes not previously identified in other catfish and revealed 1633 differentially expressed genes between challenged and control samples [222]. The pathway analysis of the differentially expressed gene set indicated the centrality of actin cytoskeletal polymerization/remodeling and junctional regulation in pathogen entry and subsequent inflammatory responses. Then, the transcriptomic profiling of host responses to columnaris was conducted in catfish, using Illumina-based RNA-seq [216]. The results revealed several central signatures following infection, including the dramatic upregulation of a rhamnose-binding lectin, NF-κB suppression and strong induction of IFN-inducible responses. As the increasing application of these new methods, greater advances will be achieved in the immunity research of catfish.

## 4. Conclusion and Future Efforts

In recent years, considerable advances have been achieved in understanding the structures and functions of innate immune genes in catfish, while some years ago these studies were only focused on model species such as zebrafish. As with the development of studies on innate immune genes, catfish has been the model species for the study of comparative immunology. Many innate immune-related genes in catfish, such as chemokines, have been studied deeply and comprehensively, and could be used as a reference in studies of other fish species.

To date, a number of relevant genes of innate immunity have been identified in catfish, and the expression analyses were conducted mostly. Aside from the genes summarized above, many immunoregulatory proteins have been extensively characterized in catfish, such as NITRs and LITRs, which are the families of putative inhibitory and stimulatory receptors found only in teleosts [223–225]. And several other immune-related genes and antigen gene have also been characterized in catfish, such as calreticulin [226], ceruloplasmin [227], cathepsin D [228], matrix metalloproteinase-13 [229], warm temperature acclimation-related 65 kDa protein (wap65) [230], pentraxin [231] and *Ichthyophthirius multifiliis*[232,233]. However, several other genes playing important roles in immune response are not identified in catfish, such as DC-specific ICAM-3 grabbing nonintegrin (DC-SIGN), which is an essential member of the C-lectin family and has been identified from zebrafish [234], and IL-1RII, which has been found in rainbow trout [18], gilthead seabream (*Sparus aurata*) [235] and Japanese flounder [236]. The immune-related genes in catfish are still in need of extensive identification.

As stated above, after being challenged with pathogenic organisms, the expressions of a large number of these genes were induced significantly, suggesting the possible roles in the innate defense response. However, the exact roles and functions during challenge remain largely unknown. Further work is warranted to study the detailed functional characterization of these genes in catfish. In addition, the effort of increasing the immunity of catfish may prove beneficial in catfish breeding and health management. Ascorbic acid has been used in the culture of striped catfish and appears to prove high level of survival rate against the artificial injection [237]. The study of infection level and fish mortality caused by *I. multifiliis* among channel catfish, blue catfish and catfish hybrids have been conducted, and no significant differences were observed [238]. Despite the progress in this productive research, the precise mechanism remains largely unknown. Further studies are needed, focused mainly on the difference between gene structure and expression.

Channel catfish, the most important cultured species in catfish, always shows resistance to infection from *E. ictaluri*. However, the blue catfish is generally considered more resistant to *E. ictaluri* infection than the channel catfish [239]. Several researches were conducted to detect the different expression profiles of innate immune genes between channel catfish and blue catfish, such as MBL [150,152]. This will present more evidences and provide insight into the different resistance between channel catfish and blue catfish. More studies are warranted to determine the different expression profiles between the two catfish species on other immune genes, including other lectins, AMPS and chemokines. Furthermore, most studies on innate immune genes in catfish focused on the channel catfish, the production of which was considerably more than the other catfish species in the USA. The study should also be carried out on other species, such as the Chinese longsnout catfish (*Leiocassis longirostris*), Japanese catfish, walking catfish and yellow catfish, which are of importance in other regions.

In the selective breeding program of catfish, growth and disease resistance are the two most important traits used by breeders [78]. Increasing disease resistance and enhancing immune system will be crucial for improving the survival rate and risk reduction in the aquaculture process. To capitalize on the variation of disease resistance, selective breeding efforts are currently underway for genetic improvement, which depends on identifying individuals with superior performance. Many innate immune genes have been found to be induced by infections, some of which are suggested to be genetic markers for disease resistance and traits to be selected as in breeding programs. Based on the immune-related genes discovered, selective breeding and transgenesis for genes that control important immune mediators have been investigated widely [240]. These efforts will allow for more rapid selective breeding and improve health conditions of catfish.

It is also noteworthy that the expressions of innate immune genes always trigger a signaling pathway associated with adaptive immunity. For example, during ESC challenge, the expression patterns of TLR5 in catfish [40] were consistent with that of B-lymphocytes [241], which was involved in activation of the adaptive immune system. Thus, determining the roles of expression of innate immune genes in adaptive immune response will provide more information and greater understanding of the relationship of innate and adaptive immune response in catfish. Concerning PRRs, although numerous advances have been achieved more recently, very little is known about the downstream signaling mechanism, exact PAMPs of individual receptors genes and the interactions among different PRRs. For example, the role NOD1 played in immune response, including sensing pathogenic organisms and signaling to induce primary immune responses, may be involved in the activation of TLRs to further guard against bacteria invasion in the intestine. At this point, further studies are needed to clarify these specific mechanisms.

To characterize the innate immune genes in catfish and understand their protective mechanisms in immunity will help us to successfully manage disease incidents in aquaculture industry. More advances will be achieved in the future, contributing to the accumulation and development of these technologies in catfish.

## Figures and Tables

**Table 1:** Summary of immune-related genes characterized from catfish.

Gene family	Gene	Species	GenBank Acc. Nos.	Reference
Toll-like receptor	TLR3	channel catfish	AY741552	[40]
	TLR5		AY741553	[40]
	TLR2		DQ372072	[41]
	TLR5S		DQ529272, DQ529273	[42]
	TLR20		DQ529274, DQ529275	[42]
	TLR21		DQ529276, DQ529277	[42]

NOD-like receptor	NOD1	channel catfish	FJ004844	[43]
	NOD2		FJ004845, JP593145	[37,43]
	NOD3a		FJ004846, JP593146	[37,43]
	NOD3b		JP593147	[37]
	NOD4		FJ004847	[43]
	NOD5		FJ004848	[43]
	NLR-B1		JP593148	[37]
	NLR-B2		JP593149	[37]
	NLR-C1		JP593150	[37]
	NLR-C2		JP593151	[37]
	NLR-C3		JP593152	[37]
	NLR-C4		JP593153	[37]
	NLR-C5		JP593154	[37]
	NLR-C6		JP593155	[37]
	NLR-C7		JP593156	[37]
	NLR-C8		JP593157	[37]
	NLR-C9		JP593158	[37]
	NLR-C10		JP593159	[37]
	NLR-C11		JP593160	[37]
	Apaf1		JP593161	[37]
	CIITA		JP593162	[37]
	NACHT-P1		JP593163	[37]

RLR-like receptor	RIG-I	channel catfish	JQ008940	[38]
	MDA5		JQ008941	[38]
	LGP2		JQ008942	[38]

Antimicrobial peptide	hepcidin	channel catfish	AY834211, AY834209	[44]
	hepcidin	blue catfish	AY834210	[44]
	LEAP-2	channel catfish	AY845143, AY845141	[45]
	LEAP-2	blue catfish	AY845142	[45]
	type1 NK-lysin	channel catfish	AY934593, AY934592, DQ153188	[23,46]
	type2 NK-lysin		DQ153189, DQ153186	[46]
	type3 NK-lysin		DQ153190, DQ153187	[46]
	BPI		AY816351	[47]

Complement	CFI	channel catfish	GQ149234	[48]
	CD59		DQ863511	[49]
	Bf/C2A		JN995600	[34]
	Bf/C2B		JN995601	[34]
	Df		JN995602	[34]

Lectin	IntL1	channel catfish	EU030378	[50]

Lectin	IntL2	channel catfish	EU030379, EU030382	[50]
	IntL1	blue catfish	EU030380	[50]
	IntL2		EU030381	[50]
	saIntL	silurus asotus	AB598141, AB598142	[51]

Chemokine	CXC	channel catfish	AY335949, AY335950	[52]
	CXC	blue catfish	AY335951	[52]
	IL-8	channel catfish	AY140803, AY140804, AY140806	[53]
	CXCL2-like		AY836754	[54]
	CXCL12		AY836755	[54]
	CXCL14		AY836756	[54]
	SCYA101	blue catfish	AY555498, DQ173276	[55,56]
	SCYA102	channel catfish	AY555499, DQ173277	[55,56]
	SCYA103	blue catfish	AY555500, DQ173278	[55,56]
	SCYA104		AY555501, DQ173279	[55,56]
	SCYA104	channel catfish	AY555512, AY555513	[55]
	SCYA105		AY555502	[55]
	SCYA106	blue catfish	AY555503, DQ173280	[55,56]
	SCYA107		AY555504, DQ173281	[55,56]
	SCYA108	channel catfish	AY555505, DQ173282	[55,56]
	SCYA109	blue catfish	AY555506, DQ173283	[55,56]
	SCYA110		AY555507, DQ173284	[55,56]
	SCYA111	channel catfish	AY555508, DQ173285	[55,56]
	SCYA112		AY555509, DQ173286	[55,56]
	SCYA113		AY555510, DQ173287	[55,56]
	SCYA114	blue catfish	AY555511, DQ173288	[55,56]
	SCYA115	channel catfish	CF263545, DQ173289	[56,57]
	SCYA116		CB940570, DQ173290	[56,57]
	SCYA117		CB939858, DQ173291	[56,57]
	SCYA118		CB939818, DQ173292	[56,57]
	SCYA119		CB939816, DQ173293	[56,57]
	SCYA120		CB939490, DQ173294	[56,57]
	SCYA121		CB939368, DQ173295	[56,57]
	SCYA122		CB937579, DQ173296	[56,57]
	SCYA123		CB937548	[57]
	SCYA124		CB937269, DQ173297	[56,57]
	SCYA125		BM028237	[57]
	SCYA126		BM027974, DQ173298	[56,57]
	CXCR4		GQ169128	[58]

Interferon	IFN-1	channel catfish	AY847297	[59]
	IFN-2		AY847295	[59]
	IFN-4		AY847296	[59]
	IFN-γ1		DQ124249	[60]
	IFN-γ2a		DQ124250	[60]
	IFN-γ2b		DQ124251	[60]

Interleukin	IL-1β gene 1	channel catfish	DQ157741, DQ160229	[61]
	IL-1β gene 2		DQ157742, DQ160230	[61]

Transferrin	transferrin	channel catfish	FJ176740, FJ176741	[62]

## References

[1] Ferraris C.J., de Pinna M.C.C. (1999). Higher-level names for catfishes (Actinopterygii: Ostariophysi: Siluriformes). Proc. Calif. Acad. Sci.

[2] Buentello J.A., Gatlin D.M., Neill W.H. (2000). Effects of water temperature and dissolved oxygen on daily feed consumption, feed utilization and growth of channel catfish (*Ictalurus punctatus*). Aquaculture.

[3] Peatman E., Terhune J., Baoprasertkul P., Xu P., Nandi S., Wang S., Somridhivej B., Kucuktas H., Li P., Dunham R. (2008). Microarray analysis of gene expression in the blue catfish liver reveals early activation of the MHC class I pathway after infection with *Edwardsiella ictaluri*. Mol. Immunol.

[4] Buurma B.J., Diana J.S. (1994). Effects of feeding frequency and handling on growth and mortality of cultured walking catfish *Clarias fuscus*. J. World Aquacult. Soc.

[5] Lefevre S., Huong D.T.T., Wang T., Phuong N.T., Bayley M. (2011). Hypoxia tolerance and partitioning of bimodal respiration in the striped catfish (*Pangasianodon hypophthalmus*). Comp. Biochem. Phys.

[6] FAO (Food and Agriculture Organization of the United Nations) http://www.fao.org/fishery/statistics/global-aquaculture-production/query/en.

[7] Yeh H.Y., Shoemaker C.A., Klesius P.H. (2005). Evaluation of a loop-mediated isothermal amplification method for rapid detection of channel catfish *Ictalurus punctatus* important bacterial pathogen *Edwardsiella ictaluri*. J. Microbiol. Meth.

[8] Crumlish M., Dung T.T., Turnbull J.F., Ngoc N.T.N., Ferguson H.W. (2002). Identification of *Edwardsiella ictaluri* from diseased freshwater catfish, *Pangasius hypophthalmus* (Sauvage), cultured in the Mekong Delta, Vietnam. J. Fish Dis.

[9] Dung T.T., Haesebrouck F., Tuan N.A., Sorgeloos P., Baele M., Decostere A. (2008). Antimicrobial susceptibility pattern of *Edwardsiella ictaluri isolates* from natural outbreaks of bacillary necrosis of *Pangasianodon hypophthalmus* in Vietnam. Microb. Drug Resist.

[10] Thinh N.H., Kuo T.Y., Hung L.T., Loc T.H., Chen S.C., Evensen O., Schuurman H.J. (2009). Combined immersion and oral vaccination of Vietnamese catfish (*Pangasianodon hypophthalmus*) confers protection against mortality caused by *Edwardsiella ictaluri*. Fish Shellfish Immunol.

[11] Dung T.T., Chiers K., Tuan N.A., Sorgeloos P., Haesebrouck F., Decostere A. (2012). Early interactions of *Edwardsiella ictaluri*, with Pangasianodon catfish and its invasive ability in cell lines. Vet. Res. Commun.

[12] Crumlish M., Thanh P.C., Koesling J., Tung V.T., Gravningen K. (2010). Experimental challenge studies in Vietnamese catfish, *Pangasianodon hypophthalmus* (Sauvage), exposed to *Edwardsiella ictaluri* and *Aeromonas hydrophila*. J. Fish Dis.

[13] Bartie K.L., Austin F.W., Diab A., Dickson C., Dung T.T., Giacomini M., Crumlish M. (2012). Intraspecific diversity of *Edwardsiella ictaluri* isolates from diseased freshwater catfish, *Pangasianodon hypophthalmus* (Sauvage), cultured in the Mekong Delta, Vietnam. J. Fish Dis.

[14] Magnadóttir B. (2006). Innate immunity of fish (overview). Fish Shellfish Immunol.

[15] Whyte S.K. (2007). The innate immune response of finfish—A review of current knowledge. Fish Shellfish Immunol.

[16] Ullal A.J., Litaker R.W., Noga E.J. (2008). Antimicrobial peptides derived from hemoglobin are expressed in epithelium of channel catfish (*Ictalurus punctatus*, Rafinesque). Dev. Comp. Immunol.

[17] Casadei E., Wang T., Zou J., Vecino J.L.G., Wadsworth S., Secombes C.J. (2009). Characterization of three novel beta-defensin antimicrobial peptides in rainbow trout (*Oncorhynchus mykiss*). Mol. Immunol.

[18] Sangrador-Vegas A., Martin S.A., O’Dea P.G., Smith T.J. (2000). Cloning and characterization of the rainbow trout (*Oncorhynchus mykiss*) type II interleukin-1 receptor cDNA. Eur. J. Biochem.

[19] Falco A., Chico V., Marroqui L., Perez L., Coll J.M., Estepa A. (2008). Expression and antiviral activity of a beta-defensin-like peptide identified in the rainbow trout (*Oncorhynchus mykiss*) EST sequences. Mol. Immunol.

[20] Wu X.Y., Xiang L.X., Huang L., Jin Y., Shao J.Z. (2008). Characterization, expression and evolution analysis of Toll-like receptor 1 gene in pufferfish (*Tetraodon nigroviridis*). Int. J. Immunogenet.

[21] Wang K.J., Cai J.J., Cai L., Qu H.D., Yang M., Zhang M. (2009). Cloning and expression of a hepcidin gene from a marine fish (*Pseudosciaena crocea*) and the antimicrobial activity of its synthetic peptide. Peptides.

[22] Xiao X., Qin Q., Chen X. (2011). Molecular characterization of a Toll-like receptor 22 homologue in large yellow croaker (*Pseudosciaena crocea*) and promoter activity analysis of its 5′-flanking sequence. Fish Shellfish Immunol.

[23] Wang Q., Bao B., Wang Y., Peatman E., Liu Z. (2006). Characterization of a NK-lysin antimicrobial peptide gene from channel catfish. Fish Shellfish Immunol.

[24] Pridgeon J.W., Russo R., Shoemaker C.A., Klesius P.H. (2010). Expression profiles of toll-like receptors in anterior kidney of channel catfish, *Ictalurus punctatus* (Rafinesque), acutely infected by *Edwardsiella ictaluri*. J. Fish Dis.

[25] Liu Z. (2011). Development of genomic resources in support of sequencing, assembly, and annotation of the catfish genome. Comp. Biochem. Phys.

[26] Lu J., Peatman E., Yang Q., Wang S., Hu Z., Reecy J., Kucuktas H., Liu Z. (2011). The catfish genome database cBARBEL: An informatic platform for genome biology of ictalurid catfish. Nucleic. Acids. Res.

[27] Clem L.W., Bly J.E., Ellsaesser C.F., Lobb C.J., Miller N.W. (1990). Channel catfish as an unconventional model for immunological studies. J. Exp. Zool. Suppl.

[28] Vallejo A.N., Miller N.W., Clem L.W. (1991). Phylogeny of immune recognition: Role of alloantigens in antigen presentation in channel catfish immune responses. Immunology.

[29] Shen L., Stuge T.B., Zhou H., Khayat M., Barker K.S., Quiniou S.M.A., Wilson M., Bengten E., Chinchar V.G., Clem L.W. (2002). Channel catfish cytotoxic cells: A mini-review. Dev. Comp. Immunol.

[30] Miller N., Wilson M., Bengten E., Stuge T., Warr G., Clem W. (1998). Functional and molecular characterization of teleost leukocytes. Immunol. Rev.

[31] Shen L., Stuge T.B., Bengten E., Wilson M., Chinchar V.G., Naftel J.P., Bernanke J.M., Clem L.W., Miller N.W. (2004). Identification and characterization of clonal NK-like cells from channel catfish (*Ictalurus punctatus*). Dev. Comp. Immunol.

[32] Liu Z. (2003). A review of catfish genomics: Progress and perspectives. Comp. Funct. Genomics.

[33] LaFrentz B.R., Shoemaker C.A., Booth N.J., Peterson B.C., Ourth D.D. (2012). Spleen index and mannose-binding lectin levels in four channel catfish families exhibiting different susceptibilities to *Flavobacterium columnare* and *Edwardsiella ictaluri*. J. Aquat. Anim. Health.

[34] Zhou Z., Liu H., Liu S., Sun F., Peatman E., Kucuktas H., Kaltenboeck L., Feng T., Zhang H., Niu D. (2012). Alternative complement pathway of channel catfish (*Ictalurus punctatus*): Molecular characterization, mapping and expression analysis of factors Bf/C2 and Df. Fish Shellfish Immunol.

[35] Zhang H., Peatman E., Liu H., Niu D., Feng T., Kucuktas H., Waldbieser G., Chen L., Liu Z. (2012). Characterization of a mannose-binding lectin from channel catfish (*Ictalurus punctatus*). Res. Vet. Sci.

[36] Pridgeon J.W., Mu X., Klesius P.H. (2012). Expression profiles of seven channel catfish antimicrobial peptides in response to *Edwardsiella ictaluri* infection. J. Fish Dis.

[37] Rajendran K.V., Zhang J., Liu S., Kucuktas H., Wang X., Liu H., Sha Z., Terhune J., Peatman E., Liu Z. (2012). Pathogen recognition receptors in channel catfish: I. Identification, phylogeny and expression of NOD-like receptors. Dev. Comp. Immunol.

[38] Rajendran K.V., Zhang J., Liu S., Peatman E., Kucuktas H., Wang X., Liu H., Wood T., Terhune J., Liu Z. (2012). Pathogen recognition receptors in channel catfish: II. Identification, phylogeny and expression of retinoic acid-inducible gene I (RIG-I)-like receptors (RLRs). Dev. Comp. Immunol.

[39] Aoki T., Takano T., Santos M.D., Kondo H., Hirono I., Tsukamoto K., Kawamura T. (2008). Molecular innate immunity in teleost fish: Review and future perspectives. Fisheries for Global Welfare and Environment, Memorial Book of the 5th World Fisheries Congress.

[40] Bilodeau A.L., Waldbieser G.C. (2005). Activation of TLR3 and TLR5 in channel catfish exposed to virulent *Edwardsiella ictaluri*. Dev. Comp. Immunol.

[41] Baoprasertkul P., Peatman E., Abernathy J., Liu Z. (2007). Structural characterisation and expression analysis of toll-like receptor 2 gene from catfish. Fish Shellfish Immunol.

[42] Baoprasertkul P., Xu P., Peatman E., Kucuktas H., Liu Z. (2007). Divergent Toll-like receptors in catfish (*Ictalurus punctatus*): TLR5S, TLR20, TLR21. Fish Shellfish Immunol.

[43] Sha Z., Abernathy J.W., Wang S., Li P., Kucuktas H., Liu H., Peatman E., Liu Z. (2009). NOD-like subfamily of the nucleotide-binding domain and leucine-rich repeat containing family receptors and their expression in channel catfish. Dev. Comp. Immunol.

[44] Bao B., Peatman E., Li P., He C., Liu Z. (2005). Catfish hepcidin gene is expressed in a wide range of tissues and exhibits tissue-specific upregulation after bacterial infection. Dev. Comp. Immunol.

[45] Bao B., Peatman E., Xu P., Li P., Zeng H., He C., Liu Z. (2006). The catfish liver-expressed antimicrobial peptide 2 (LEAP-2) gene is expressed in a wide range of tissues and developmentally regulated. Mol. Immunol.

[46] Wang Q., Wang Y., Xu P., Liu Z. (2006). NK-lysin of channel catfish: Gene triplication, sequence variation, and expression analysis. Mol. Immunol.

[47] Xu P., Bao B., He Q., Peatman E., He C., Liu Z. (2005). Characterization and expression analysis of bactericidal permeability-increasing protein (BPI) antimicrobial peptide gene from channel catfish *Ictalurus punctatus*. Dev. Comp. Immunol.

[48] Abernathy J.W., Lu J., Liu H., Kucuktas H., Liu Z. (2009). Molecular characterization of complement factor I reveals constitutive expression in channel catfish. Fish Shellfish Immunol.

[49] Yeh H.Y., Klesius P.H. (2007). Molecular cloning and expression of channel catfish, *Ictalurus punctatus*, complement membrane attack complex inhibitor CD59. Vet. Immunol. Immunop.

[50] Takano T., Sha Z., Peatman E., Terhune J., Liu H., Kucuktas H., Li P., Edholm E.S., Wilson M., Liu Z. (2008). The two channel catfish intelectin genes exhibit highly differential patterns of tissue expression and regulation after infection with *Edwardsiella ictaluri*. Dev. Comp. Immunol.

[51] Tsutsui S., Komatsu Y., Sugiura T., Araki K., Nakamura O. (2011). A unique epidermal mucus lectin identified from catfish (*Silurus asotus*): First evidence of intelectin in fish skin slime. J. Biochem.

[52] Baoprasertkul P., Peatman E., Chen L., He C., Kucuktas H., Li P., Simmons M., Liu Z. (2004). Sequence analysis and expression of a CXC chemokine in resistant and susceptible catfish after infection of *Edwardsiella ictaluri*. Dev. Comp. Immunol.

[53] Chen L., He C., Baoprasertkul P., Xu P., Li P., Serapion J., Waldbieser G., Wolters W., Liu Z. (2005). Analysis of a catfish gene resembling interleukin-8: cDNA cloning, gene structure, and expression after infection with *Edwardsiella ictaluri*. Dev. Comp. Immunol.

[54] Baoprasertkul P., He C., Peatman E., Zhang S., Li P., Liu Z. (2005). Constitutive expression of three novel catfish CXC chemokines: Homeostatic chemokines in teleost fish. Mol. Immunol.

[55] He C., Peatman E., Baoprasertkul P., Kucuktas H., Liu Z. (2004). Multiple CC chemokines in channel catfish and blue catfish as revealed by analysis of expressed sequence tags. Immunogenetics.

[56] Bao B., Peatman E., Peng X., Baoprasertkul P., Wang G., Liu Z. (2006). Characterization of 23 CC chemokine genes and analysis of their expression in channel catfish (*Ictalurus punctatus*). Dev. Comp. Immunol.

[57] Peatman E., Bao B., Baoprasertkul P., Liu Z. (2005). *In silico* identification and expression analysis of 12 novel CC chemokines in catfish. Immunogenetics.

[58] Yeh H.Y., Klesius P.H. (2010). Sequence analysis, characterization and mRNA distribution of channel catfish (*Ictalurus punctatus* Rafinesque, 1818) chemokine (C-X-C motif) receptor 4 (CXCR4) cDNA. Vet. Immunol. Immunop.

[59] Long S., Milev-Milovanovic I., Wilson M., Bengten E., Clem L.W., Miller N.W., Chinchar V.G. (2006). Identification and expression analysis of cDNAs encoding channel catfish type I interferons. Fish Shellfish Immunol.

[60] Milev-Milovanovic I., Long S., Wilson M., Bengten E., Miller N.W., Chinchar V.G. (2006). Identification and expression analysis of interferon gamma genes in channel catfish. Immunogenetics.

[61] Wang Y., Wang Q., Baoprasertkul P., Peatman E., Liu Z. (2006). Genomic organization, gene duplication, and expression analysis of interleukin-1beta in channel catfish (*Ictalurus punctatus*). Mol. Immunol.

[62] Liu H., Takano T., Abernathy J., Wang S., Sha Z., Jiang Y., Terhune J., Kucuktas H., Peatman E., Liu Z. (2010). Structure and expression of transferrin gene of channel catfish, *Ictalurus punctatus*. Fish Shellfish Immunol.

[63] Medzhitov R., Janeway J.C. (2000). Innate immune recognition: Mechanisms and pathways. Immunol. Rev.

[64] Janeway J.C.A., Medzhitov R. (2002). Innate immune recognition. Annu. Rev. Immunol.

[65] Takeuchi O., Akira S. (2010). Pattern recognition receptors and inflammation. Cell.

[66] Jault C., Pichon L., Chluba J. (2004). Toll-like receptor gene family and TIR-domain adapters in *Danio rerio*. Mol. Immunol.

[67] Meijer A.H., Gabby Krens S.F., Medina Rodriguez I.A., He S., Bitter W., Ewa Snaar-Jagalska B., Spaink H.P. (2004). Expression analysis of the Toll-like receptor and TIR domain adaptor families of zebrafish. Mol. Immunol.

[68] Tsujita T., Tsukada H., Nakao M., Oshiumi H., Matsumoto M., Seya T. (2004). Sensing bacterial flagellin by membrane and soluble orthologs of Toll-like receptor 5 in rainbow trout (*Onchorhynchus mikiss*). J. Biol. Chem.

[69] Palti Y., Gahr S.A., Purcell M.K., Hadidi S., Rexroad C.E., Wiens G.D. (2010). Identification, characterization and genetic mapping of TLR7, TLR8a1 and TLR8a2 genes in rainbow trout (*Oncorhynchus mykiss*). Dev. Comp. Immunol.

[70] Kongchum P., Palti Y., Hallerman E.M., Hulata G., David L. (2010). SNP discovery and development of genetic markers for mapping innate immune response genes in common carp (*Cyprinus carpio*). Fish Shellfish Immunol.

[71] Oshiumi H., Tsujita T., Shida K., Matsumoto M., Ikeo K., Seya T. (2003). Prediction of the prototype of the human Toll-like receptor gene family from the pufferfish, *Fugu rubripes*, genome. Immunogenetics.

[72] Rebl A., Siegl E., Köllner B., Fischer U., Seyfert H.M. (2007). Characterization of twin toll-like receptors from rainbow trout (*Oncorhynchus mykiss*): Evolutionary relationship and induced expression by *Aeromonas salmonicida salmonicida*. Dev. Comp. Immunol.

[73] Laing K.J., Purcell M.K., Winton J.R., Hansen J.D. (2008). A genomic view of the NOD-like receptor family in teleost fish: Identification of a novel NLR subfamily in zebrafish. BMC Evol. Biol.

[74] Hansen J.D., Vojtech L.N., Laing K.J. (2011). Sensing disease and danger: A survey of vertebrate PRRs and their origins. Dev. Comp. Immunol.

[75] Akira S., Uematsu S., Takeuchi O. (2006). Pathogen recognition and innate immunity. Cell.

[76] Beutler B. (2004). Inferences, questions and possibilities in toll-like receptor signalling. Nature.

[77] Bilodeau-Bourgeois L., Bosworth B.G., Peterson B.C. (2008). Differences in mortality, growth, lysozyme, and toll-like receptor gene expression among genetic groups of catfish exposed to virulent *Edwardsiella ictaluri*. Fish Shellfish Immunol.

[78] Peterson B.C., Bosworth B.G., Bilodeau A.L. (2005). Differential gene expression of IGF-I, IGF-II, and toll-like receptors 3 and 5 during embryogenesis in hybrid (channel × blue) and channel catfish. Comp. Biochem. Phys.

[79] Bilodeau A.L., Peterson B.C., Bosworth B.G. (2006). Response of toll-like receptors, lysozyme, and IGF-I in back-cross hybrid (F1 male (blue × channel) × female channel) catfish challenged with virulent *Edwardsiella ictaluri*. Fish Shellfish Immunol.

[80] Baoprasertkul P., Peatman E., Somridhivej B., Liu Z. (2006). Toll-like receptor 3 and TICAM genes in catfish: Species-specific expression profiles following infection with *Edwardsiella ictaluri*. Immunogenetics.

[81] Carneiro L., Magalhaes J., Tattoli I., Philpott D., Travassos L. (2008). NOD-like proteins in inflammation and disease. J. Pathol.

[82] Rosenstiel P., Jacobs G., Till A., Schreiber S. (2008). NOD-like receptors: Ancient sentinels of the innate immune system. Cell. Mol. Life Sci.

[83] Takeuchi O., Akira S. (2008). MDA5/RIG-I and virus recognition. Curr. Opin. Immunol.

[84] Kawai T., Akira S. (2009). The roles of TLRs, RLRs and NLRs in pathogen recognition. Int. Immunol.

[85] Yoneyama M., Kikuchi M., Matsumoto K., Imaizumi T., Miyagishi M., Taira K., Foy E., Loo Y.M., Gale M., Akira S. (2005). Shared and unique functions of the DExD/H-box helicases RIG-I, MDA5, and LGP2 in antiviral innate immunity. J. Immunol..

[86] Lauksund S., Svingerud T., Bergan V., Robertsen B. (2009). Atlantic salmon IPS-1mediates induction of IFNa1 and activation of NF-kB and localizes to mitochondria. Dev. Comp. Immunol.

[87] Huang T., Su J., Heng J., Dong J., Zhang R., Zhu H. (2010). Identification and expression profiling analysis of grass carp *Ctenopharyngodon idella* LGP2 cDNA. Fish Shellfish Immunol.

[88] Ohtani M., Hikima J., Kondo H., Hirono I., Jung T.S., Aoki T. (2010). Evolutional conservation of molecular structure and antiviral function of a viral RNA receptor, LGP2, in Japanese Flounder, *Paralichthys olivaceus*. J. Immunol.

[89] Su J., Huang T., Dong J., Heng J., Zhang R., Peng L. (2010). Molecular cloning and immune responsive expression of MDA5 gene, a pivotal member of the RLR gene family from grass carp *Ctenopharyngodon idella*. Fish Shellfish Immunol.

[90] Yang C., Su J., Huang T., Zhang R., Peng L. (2011). Identification of a retinoic acidinducible gene I from grass carp (*Ctenopharyngodon idella*) and expression analysis *in vivo* and *in vitro*. Fish Shellfish Immunol.

[91] Su Y. (2011). Isolation and identification of pelteobagrin, a novel antimicrobial peptide from the skin mucus of yellow catfish (*Pelteobagrus fulvidraco*). Comp. Biochem. Phys.

[92] Bowdish D.M.E., Davidson D.J., Scott M.G., Hancock R.E.W. (2005). Immunomodulatory activities of small host defense peptides. Antimicrob. Agents Ch.

[93] Niyonsaba F., Ushio H., Hara M., Yokoi H., Tominaga M., Takamori K., Kajiwara N., Saito H., Nagaoka I., Ogawa H. (2010). Antimicrobial peptides human beta-defensins and cathelicidin LL-37 induce the secretion of a pruritogenic cytokine IL-31 by human mast cells. J. Immunol.

[94] Shike H., Lauth X., Westerman M.E., Ostland V.E., Carlberg J.M., van Olst J.C., Shimizu C., Bulet P., Burns J.C. (2002). Bass hepcidin is a novel antimicrobial peptide induced by bacterial challenge. Eur. J. Biochem.

[95] Shike H., Shimizu C., Lauth X., Burns J.C. (2004). Organization and expression analysis of the zebrafish hepcidin gene, an antimicrobial peptide gene conserved among vertebrates. Dev. Comp. Immunol.

[96] Noga E.J., Silphaduang U. (2003). Piscidins: A novel family of peptide antibiotics from fish. Drug News Perspect.

[97] Douglas S.E., Gallant J.W., Liebscher R.S., Dacanay A., Tsoi S.C. (2003). Identification and expression analysis of hepcidin-like antimicrobial peptides in bony fish. Dev. Comp. Immunol.

[98] Douglas S.E., Patrzykat A., Pytyck J., Gallant J.W. (2003). Identification, structure and differential expression of novel pleurocidins clustered on the genome of the winter flounder, *Pseudopleuronectes americanus* (Walbaum). Eur. J. Biochem.

[99] Zhang Y.A., Zou J., Chang C.I., Secombes C.J. (2004). Discovery and characterization of two types of liver-expressed antimicrobial peptide 2 (LEAP-2) genes in rainbow trout. Vet. Immunol. Immunop.

[100] Zhou J.G., Wei J.G., Xu D.H., Cui H.C., Yan Y., Ou-Yang Z.L., Huang X.H., Huang Y.H., Qin Q.W. (2011). Molecular cloning and characterization of two novel hepcidins from orange-spotted grouper, *Epinephelus coioides*. Fish Shellfish Immunol.

[101] Robinette D., Wada S., Arroll T., Levy M.G., Miller W.L., Noga E.J. (1998). Antimicrobial activity in the skin of the channel catfish *Ictalurus punctatus*: Characterization of broad-spectrum histone-like antimicrobial proteins. Cell. Mol. Life Sci.

[102] Cho J.H., Park I.Y., Kim H.S., Lee W.T., Kim M.S., Kim S.C. (2002). Cathepsin D produces antimicrobial peptide parasin I from histone H2A in the skin mucosa of fish. FASEB J.

[103] Park I.Y., Park C.B., Kim M.S., Kim S.C. (1998). Parasin I, an antimicrobial peptide derived from histone H2A in the catfish, *Parasilurus asotus*. FEBS Lett.

[104] Noga E.J., Ullal A.J., Corrales J., Fernandes J.M.O. (2011). Application of antimicrobial polypeptide host defenses to aquaculture: Exploitation of downregulation and upregulation responses. Comp. Biochem. Phys.

[105] Zahran E., Seo J.K., Noga E.J. (2012). The effect of adjuvant and microbial challenge on the expression of antimicrobial polypeptides in channel catfish (*Ictalurus punctatus*). Fish Shellfish Immunol.

[106] Marel M., Adamek M., Gonzalez S.F., Frost P., Rombout J.H., Wiegertjes G.F., Savelkoul H.F., Steinhagen D. (2012). Molecular cloning and expression of two β-defensin and two mucin genes in common carp (*Cyprinus carpio* L.) and their up-regulation after β-glucan feeding. Fish Shellfish Immunol.

[107] Zou J., Mercier C., Koussounadis A., Secombes C. (2007). Discovery of multiple β-defensin like homologues in teleost fish. Mol. Immunol.

[108] Nam B.H., Moon J.Y., Kim Y.O., Kong H.J., Kim W.J., Lee S.J., Kim K.K. (2010). Multiple β-defensin isoforms identified in early developmental stages of the teleost *Paralichthys olivaceus*. Fish Shellfish Immunol.

[109] Zhao J.G., Zhou L., Jin J.Y., Zhao Z., Lan J., Zhang Y.B., Zhang Q.Y., Gui J.F. (2009). Antimicrobial activity-specific to Gram-negative bacteria and immune modulation-mediated NF-κB and Sp1 of a medaka β-defensin. Dev. Comp. Immunol.

[110] Jin J.Y., Zhou L., Wang Y., Li Z., Zhao J.G., Zhang Q.Y., Gui J.F. (2010). Antibacterial and antiviral roles of a fish β-defensin expressed both in pituitary and testis. PLoS One.

[111] Saeij J.P., Wiegertjes G.F., Stet R.J. (1999). Identification and characterization of a fish natural resistance-associated macrophage protein (NRAMP) cDNA. Immunogenetics.

[112] Chen S.L., Zhang Y.X., Xu J.Y., Meng L., Sha Z., Ren G.C. (2007). Molecular cloning, characterization and expression analysis of natural resistance associated macrophage protein (Nramp) cDNA from turbot (*Scophthalmus maximus*). Comp. Biochem. Phys.

[113] Chen S.L., Wang Z.J., Xu M.Y., Gui J.F. (2006). Molecular identification and expression analysis of natural resistance associated macrophage protein (Nramp) cDNA from Japanese flounder (*Paralichthys olivaceus*). Fish Shellfish Immunol.

[114] Holland M.C.H., Lambris J.D. (2002). The complement system in teleosts. Fish Shellfish Immunol.

[115] Boshra H., Li J., Sunyer J.O. (2006). Recent advances on the complement system of teleost fish. Fish Shellfish Immunol.

[116] Dodds A.W., Petry F. (1993). The phylogeny and evolution of the first component of complement, C1. Behring Inst. Mitt.

[117] Hu Y.L., Pan X.M., Xiang L.X., Shao J.Z. (2010). Characterization of C1q in teleosts: Insight into the molecular and functional evolution of C1q family and classical pathway. J. Biol. Chem.

[118] Lao H.H., Sun Y.N., Yin Z.X., Wang J., Chen C., Weng S.P., He W., Guo C.J., Huang X.D., Yu X.Q. (2008). Molecular cloning of two C1q-like cDNAs in mandarin fish *Siniperca chuatsi*. Vet. Immunol. Immunop.

[119] Jenkins J.A., Rosell R., Ourth D.D., Coons L.B. (1991). Electron microscopy of bactericidal effects produced by the alternative complement pathway of channel catfish. J. Aquat. Anim. Health.

[120] Jenkins J.A., Ourth D.D. (1993). Opsonic effect of the alternative complement pathway on channel catfish peripheral blood phagocytes. Vet. Immunol. Immunop.

[121] Jenkins J.A., Ourth D.D. (1990). Membrane damage to *Escherichia coli* and bactericidal kinetics by the alternative complement pathway of channel catfish. Comp. Biochem. Phys.

[122] Ourth D.D., Machinski L.M. (1987). Bacterial sialic acid modulates activation of the alternative complement pathway of channel catfish (*Ictalurus punctatus*). Dev. Comp. Immunol.

[123] Ourth D.D., Wilson E.A. (1982). Alternate pathway of complement and bactericidal response of the channel catfish to *Salmonella paratyphi*. Dev. Comp. Immunol.

[124] Dodds A.W., Smith S.L., Levine R.P., Willis A.C. (1998). Isolation and initial characterisation of complement components C3 and C4 of the nurse shark and the channel catfish. Dev. Comp. Immunol.

[125] Cole D.S., Morgan B.P. (2003). Beyond lysis: How complement influences cell fate. Clin. Sci.

[126] Mollnes T.E., Song W.C., Lambris J.D. (2002). Complement in inflammatory tissue damage and disease. Trends Immunol.

[127] Wei W., Wu H., Xu H., Zhang X., Chu T., Wu C., Chang K., Zhang Y. (2010). Cloning and molecular characterization of lycC1INH genes in large yellow croaker (*Pseudosciaena crocea*). Fish Shellfish Immunol.

[128] Liu G., Zhang J., Chen X. (2007). Molecular and functional characterization of a CD59 analogue from large yellow croaker *Pseudosciana crocea*. Mol. Immunol.

[129] Papanastasiou A.D., Georgaka E., Zarkadis I.K. (2007). Cloning of a CD59-like gene in rainbow trout: Expression and phylogenetic analysis of two isoforms. Mol. Immunol.

[130] Ellingsen T., Strand C., Monsen E., Bogwald J., Dalmo R.A. (2005). The ontogeny of complement component C3 in the spotted wolffish (*Anarhichas minor* Olafsen). Fish Shellfish Immunol.

[131] Løvoll M., Kilvik T., Boshra H., Bogwald J., Sunyer J.O., Dalmo R.A. (2006). Maternal transfer of complement components C3-1, C3-3, C3-4, C4, C5, C7, Bf, and Df to offspring in rainbow trout (*Oncorhynchus mykiss*). Immunogenetics.

[132] Huttenhuis H.B., Grou C.P., Taverne-Thiele A.J., Taverne N., Rombout J.H. (2006). Carp (*Cyprinus carpio L*.) innate immune factors are present before hatching. Fish Shellfish Immunol.

[133] Shen Y.B., Zhang J.B., Xu X.Y., Li J.L. (2011). Molecular cloning, characterization and expression analysis of the complement component C6 gene in grass carp. Vet. Immunol. Immunop.

[134] Løvoll M., Johnsen H., Boshra H., Bogwald J., Sunyer J.O., Dalmo R.A. (2007). The ontogeny and extrahepatic expression of complement factor C3 in Atlantic salmon (*Salmo salar*). Fish Shellfish Immunol.

[135] Wang Z., Zhang S., Wang G., An Y. (2008). Complement activity in the egg cytosol of zebrafish *Danio rerio*: Evidence for the defense role of maternal complement components. PLoS One.

[136] Zhu L.Y., Nie L., Zhu G., Xiang L.X., Shao J.Z. (2012). Advances in research of fish immune-relevant genes: A comparative overview of innate and adaptive immunity in teleosts. Dev. Comp. Immunol..

[137] Saito T., Hatada M., Iwanaga S., Kawabata S. (1997). A newly identified horseshoe crab lectin with binding specificity to O-antigen of bacterial lipopolysaccharides. J. Biol. Chem.

[138] Tasumi S., Ohira T., Kawazoe I., Suetake H., Suzuki Y., Aida K. (2002). Primary structure and characteristics of a lectin from skin mucus of the Japanese eel (*Anguilla japonica*). J. Biol. Chem.

[139] Nauta A.J., Castellano G., Xu W., Woltman A.M., Borrias M.C., Daha M.R., van Kooten C., Roos A. (2004). Opsonization with C1q and mannose-binding lectin targets apoptotic cells to dendritic cells. J. Immunol.

[140] Wolfe K.G., Plumb J.A., Morrison E.E. (1998). Lectin binding characteristics of the olfactory mucosa of channel catfish: Potential factors in attachment of *Edwardsiella ictaluri*. J. Aquat. Anim. Health.

[141] Tateno H., Ogawa T., Muramoto K., Kamiya H., Saneyoshi M. (2002). Rhamnose-binding lectins from steelhead trout (*Onchorhynchus mykiss*) eggs recognize bacterial lipopolysaccharides and lipoteichoic acid. Biosci. Biotechnol. Biochem.

[142] Sharon N. (1993). Lectin-carbohydrate complexes of plants and animals: An atomic view. Trends Biochem. Sci.

[143] Drickamer K., Taylor M.E. (1993). Biology of animal lectins. Annu. Rev. Cell Biol.

[144] Tsuji S., Uehori J., Matsumoto M., Suzuki Y., Matsuhisa A., Toyoshima K., Seya T. (2001). Human intelectin is a novel soluble lectin that recognizes galactofuranose in carbohydrate chains of bacterial cell wall. J. Biol. Chem.

[145] Vasta G.R., Nita-Lazar M., Giomarelli B., Ahmed H., Du S., Cammarata M., Parrinello N., Bianchet M.A., Amzel L.M. (2011). Structural and functional diversity of the lectin repertoire in teleost fish: Relevance to innate and adaptive immunity. Dev. Comp. Immunol.

[146] Jensen L.E., Hiney M.P., Shields D.C., Uhlar C.M., Lindsay A.J., Whitehead A.S. (1997). Acute phase protein in salmonids—Evolutionary analyses and acute phase response. J. Immunol.

[147] Ottinger C.A., Johnson S.C., Ewart K.V., Brown L.L., Ross N.W. (1999). Enhancement of anti-Aeromonas salmonicida activity in Atlantic salmon (*Salmo salar*) macrophages by a mannose binding lectin. Comp. Biochem. Phys.

[148] Dong C.H., Yang S.T., Yang Z.A., Zhang L., Gui J.F. (2004). A C-type lectin associated and translocated with cortical granules during oocyte maturation and egg fertilization in fish. Dev. Biol.

[149] Tasumi S., Yang W.J., Usami T., Tsutsui S., Ohira T., Kawazoe I., Wilder M.N., Aida K., Suzuki Y. (2004). Characteristics and primary structure of a galectin in the skin mucus of the Japanese eel (*Anguilla japonica*). Dev. Comp. Immunol.

[150] Ourth D.D., Rose W.M. (2011). Purification, characterization and seasonal variation of mannose-binding C-type lectin in Ictalurid catfish. Aquaculture.

[151] Teillet F., Dublet B., Andrieu J.P., Gaboriaud C., Arlaud G.J., Thielens N.M. (2005). The two major oligomeric forms of human mannan-binding lectin: Chemical characterization, carbohydrate-binding properties, and interaction with MBL-associated serine proteases. J. Immunol.

[152] Ourth D.D., Narra M.B., Simco B.A. (2007). Comparative study of mannose-binding C-type lectin isolated from channel catfish (*Ictalurus punctatus*) and blue catfish (*Ictalurus furcatus*). Fish Shellfish Immunol.

[153] Zhang H., Peatman E., Liu H., Feng T., Chen L., Liu Z. (2012). Molecular characterization of three l-type lectin genes from channel catfish, *Ictalurus punctatus* and their responses to *Edwardsiella ictaluri* challenge. Fish Shellfish Immunol.

[154] Dutta S., Sinha B., Bhattacharya B., Chatterjee B., Mazumder S. (2005). Characterization of a galactose binding serum lectin from the Indian catfish, *Clarias batrachus*: Possible involvement of fish lectins in differential recognition of pathogens. Comp. Biochem. Phys.

[155] Singha B., Adhya M., Chatterjee B.P. (2008). Catfish (*Clarias batrachus*) serum lectin recognizes polyvalent Tn [α-d-Gal*p*NAc1-Ser/Thr], Tα [β-d-Gal*p*-(1→3)-α-d-Gal*p*NAc1-Ser/Thr], and II [β-d-Galp(1→4)-β-d-Glc*p*NAc1-] mammalian glycotopes. Carbohyd. Res.

[156] Niu D., Peatman E., Liu H., Lu J., Kucuktas H., Liu S., Sun F., Zhang H., Feng T., Zhou Z. (2011). Microfibrillar-associated protein 4 (MFAP4) genes in catfish play a novel role in innate immune responses. Dev. Comp. Immunol.

[157] Komiya T., Tanigawa Y., Hirohashi S. (1998). Cloning of the novel gene intelectin, which is expressed in intestinal paneth cells in mice. Biochem. Biophs. Res. Commun.

[158] Hosono M., Kawauchi H., Nitta K., Takayanagi Y., Shiokawa H., Mineki R., Murayama K. (1993). Purification and characterization of *Silurus asotus* (catfish) roe lectin. Biol. Pharm. Bull.

[159] Hosono M., Ishikawa K., Mineki R., Murayama K., Numata C., Ogawa Y., Takayanagi Y., Nitta K. (1999). Tandem repeat structure of rhamnose-binding lectin from catfish (*Silurus asotus*) eggs. Biochim. Biophys. Acta.

[160] Sugawara S., Sasaki S., Ogawa Y., Hosono M., Nitta K. (2005). Catfish (*Silurus asotus*) lectin enhances the cytotoxic effects of doxorubicin. Yakugaku. Zasshi.

[161] Sugawara S., Hosono M., Ogawa Y., Takayanagi M., Nitta K. (2005). Catfish egg lectin causes rapid activation of multidrug resistance 1 p-glycoprotein as a lipid translocase. Biol. Pharm. Bull.

[162] Kawano T., Sugawara S., Hosono M., Tatsuta T., Ogawa Y., Fujimura T., Taka H., Murayama K., Nitta K. (2009). Globotriaosylceramide-expressing burkitt’s lymphoma cells are committed to early apoptotic status by rhamnose-binding lectin from catfish eggs. Biol. Pharm. Bull.

[163] Neville L.F., Mathiak G., Bagasra O. (1997). The immunobiology of interferon-gamma inducible protein 10 kD (IP-10): A novel, pleiotropic member of the C-X-C chemokine superfamily. Cytokine Growth Factor Rev.

[164] Bacon K., Baggiolini M., Broxmeyer H., Horuk R., Lindley I., Mantovani A. (2002). Chemokine/chemokine receptor nomenclature. J. Interf. Cytok. Res.

[165] DeVries M.E., Kelvin A.A., Xu L., Ran L., Robinson J., Kelvin D.J. (2006). Defining the origins and evolution of the chemokine/chemokine receptor system. J. Immunol.

[166] Ransohoff R.M. (2009). Chemokines and chemokine receptors: Standing at the crossroads of immunobiology and neurobiology. Immunity.

[167] Peatman E., Liu Z. (2006). CC chemokines in zebrafish: Evidence for extensive intrachromosomal gene duplications. Genomics.

[168] Peatman E., Liu Z. (2007). Evolution of CC chemokines in teleost fish: A case study in gene duplication and implications for immune diversity. Immunogenetics.

[169] Alejo A., Tafalla C. (2011). Chemokines in teleost fish species. Dev. Comp. Immunol.

[170] Lee E.Y., Park H.H., Kim Y.T., Choi T.J. (2001). Cloning and sequence analysis of the interleukin-8 gene from flounder (*Paralichthys olivaceous*). Gene.

[171] Peatman E., Bao B., Peng X., Baoprasertkul P., Brady Y., Liu Z. (2006). Catfish CC chemokines: Genomic clustering, duplications, and expression after bacterial infection with *Edwardsiella ictaluri*. Mol. Genet. Genomics.

[172] Pestka S., Langer J.A., Zoon K.C., Samuel C.E. (1987). Interferons and their actions. Annu. Rev. Biochem.

[173] Samuel C.E. (2001). Antiviral actions of interferons. Clin. Microbiol. Rev.

[174] Zou J., Secombes C.J. (2011). Teleost fish interferons and their role in immunity. Dev. Comp. Immunol.

[175] Robertsen B. (2006). The interferon system of teleost fish. Fish Shellfish Immunol.

[176] Biron C.A., Sen G.C., Knipe D.M., Howley P.M. (2001). Fields of Virology.

[177] Aggad D., Mazel M., Boudinot P., Mogensen K.E., Hamming O.J., Hartmann R., Kotenko S., Herbomel P., Lutfalla G., Levraud J.P. (2009). The two groups of zebrafish virus-induced interferons signal via distinct receptors with specific and shared chains. J. Immunol.

[178] Sun B., Robertsen B., Wang Z., Liu B. (2009). Identification of an Atlantic salmon IFN multigene cluster encoding three IFN subtypes with very different expression properties. Dev. Comp. Immunol.

[179] Long S. (2004). Identification of a cDNA encoding channel catfish interferon. Dev. Comp. Immunol.

[180] Dinarello C.A. (1998). Interleukin-1, interleukin-1 receptors and interleukin-1 receptor agonists. Int. Rev. Immunol.

[181] Dinarello C.A. (1998). Interleukin-1β, interleukin-18, and the interleukin-1β converting enzyme. Ann. Ny. Acad. Sci.

[182] Mathew J.A., Guo Y.X., Goh K.P., Chan J., Verburg-van K.B.M., Kwang J. (2002). Characterisation of a monoclonal antibody to carp IL-1beta and the development of a sensitive capture ELISA. Fish Shellfish Immunol.

[183] Zou J., Grabowski P.S., Cunningham C., Secombes C.J. (1999). Molecular cloning of interleukin 1β from rainbow trout *Oncorhynchus mykiss* reveals no evidence of an ice cut site. Cytokine.

[184] Fujiki K., Shin D.H., Nakao M., Yano T. (2000). Molecular cloning and expression analysis of carp (*Cyprinus carpio*) interleukin-1β, high affinity immunoglobulin E Fc receptor γ subunit and serum amyloid A. Fish Shellfish Immunol.

[185] Scapigliati G., Buonocore F., Bird S., Zou J., Pelegrin P., Falasca C., Prugnoli D., Secombes C.J. (2001). Phylogeny of cytokines: Molecular cloning and expression analysis of sea bass *Dicentrarchus labrax* interleukin-1β. Fish Shellfish Immunol.

[186] Chistiakov D.A., Kabanov F.V., Troepolskaya O.D., Tischenko M.M. (2010). A variant of the interleukin-1beta gene in European sea bass, *Dicentrarchus labrax* L., is associated with increased resistance against *Vibrio anguillarum*. J. Fish Dis.

[187] Jiang S., Zhang D., Li J., Liu Z. (2008). Molecular characterization, recombinant expression and bioactivity analysis of the interleukin-1β from the yellowfin sea bream, *Acanthopagrus latus* (Houttuyn). Fish Shellfish Immunol.

[188] Locksley R.M., Killeen N., Lenardo M.J. (2001). The TNF and TNF receptor superfamilies: Integrating mammalian biology. Cell.

[189] Xiao J., Zhou Z.C., Chen C., Huo W.L., Yin Z.X., Weng S.P., Chan S.M., Yu X.Q., He J.G. (2007). Tumor necrosis factor-α gene from mandarin fish, *Siniperca chuatsi*: Molecular cloning, cytotoxicity analysis and expression profile. Mol. Immunol.

[190] Savan R., Kono T., Igawa D., Sakai M. (2005). A novel tumor necrosis factor (TNF) gene present in tandem with the TNF-α gene on the same chromosome in teleosts. Immunogenetics.

[191] Saeij J.P., Stet R.J., Vries B.J., Muiswinkel W.B., Wiegertjes G.F. (2003). Molecular and functional characterization of carp TNF: A link between TNF polymorphism and trypanotolerance. Dev. Comp. Immunol.

[192] Ordas M.C., Costa M.M., Roca F.J., Lopez-Castejon G., Mulero V., Meseguer J., Figueras A., Novoa B. (2007). Turbot TNFα gene: Molecular characterization and biological activity of the recombinant protein. Mol. Immunol.

[193] Zou J., Secombes C.J., Long S., Miller N., Clem L.W., Chinchar V.G. (2003). Molecular identification and expression analysis of tumor necrosis factor in channel catfish (*Ictalurus punctatus*). Dev. Comp. Immunol.

[194] Booth N.J., Peterson B.C. (2008). Tlr5, Nramp, TNF, and hepcidin response to challenge with *Edwardsiella ictaluri* in channel catfish families with high and low susceptibility to infection. Aquaculture.

[195] Aisen P. (1980). Iron transport and storage proteins. Annu. Rev. Biochem.

[196] Crichton R.R., Charloteaux-Wauters M. (1987). Iron transport and storage. Eur. J. Biochem.

[197] Anderson G.J., Frazer D.M. (2005). Hepatic iron metabolism. Semin. Liver. Dis.

[198] Uribe C., Folch H., Enriquez R., Moran G. (2011). Innate and adaptive immunity in teleost fish: A review. Vet. Med.

[199] Sriboonsan A., Poompuang S., Panprommin D., Areechon N., Srisapoome P. Cloning, characterization of complementary DNA and expression of transferrin gene of Gunther’s walking catfish (*Clarias macrocephalus* Gunther).

[200] Liu H., Takano T., Peatman E., Abernathy J., Wang S., Sha Z., Kucuktas H., Xu D.H., Klesius P., Liu Z. (2010). Molecular characterization and gene expression of the channel catfish ferritin H subunit after bacterial infection and iron treatment. J. Exp. Zool.

[201] Bates S. (2011). The role of gene expression profiling in drug discovery. Curr. Opin. Pharmacol.

[202] Malone J.H., Oliver B. (2011). Microarrays, deep sequencing and the true measure of the transcriptome. BMC Biology.

[203] Kai T., Williams D., Spradling A.C. (2005). The expression profile of purified *Drosophila germline* stem cells. Dev. Biol.

[204] Chan E.T., Quon G.T., Chua G., Babak T., Trochesset M., Zirngibl R.A., Aubin J., Ratcliffe M.J., Wilde A., Brudno M. (2009). Conservation of core gene expression in vertebrate tissues. J. Biol.

[205] Arbeitman M.N., Furlong E.E., Imam F., Johnson E., Null B.H., Baker B.S., Krasnow M.A., Scott M.P., Davis R.W., White K.P. (2002). Gene expression during the life cycle of *Drosophila melanogaster*. Science.

[206] Spellman P.T., Sherlock G., Zhang M.Q., Iyer V.R., Anders K., Eisen M.B., Brown P.O., Botstein D., Futcher B. (1998). Comprehensive identification of cell cycle-regulated genes of the yeast *Saccharomyces cerevisiae* by microarray hybridization. Mol. Biol. Cell.

[207] Villeneuve D.L., Wang R.L., Bencic D.C., Biales A.D., Martinović D., Lazorchak J.M., Toth G., Ankley G.T. (2009). Altered gene expression in the brain and ovaries of zebrafish (*Danio Rerio*) exposed to the aromatase inhibitor fadrozole: Microarray analysis and hypothesis generation. Environ. Toxicol. Chem.

[208] Boxtel A.L., Kamstra J.H., Cenijn P.H., Pieterse B., Wagner M.J., Antink M., Krab K., Burg B., Marsh G., Brouwer A. (2008). Microarray analysis reveals a mechanism of phenolic polybrominated diphenylether toxicity in zebrafish. Environ. Sci. Technol.

[209] Park K.C., Osborne J.A., Montes A., Dios S., Nerland A.H., Novoa B., Figueras A., Brown L.L., Johnson S.C. (2009). Immunological responses of turbot (*Psetta maxima*) to nodavirus infection or polyriboinosinic polyribocytidylic acid (pIC) stimulation, using expressed sequence tags (ESTs) analysis and cDNA microarrays. Fish Shellfish Immunol.

[210] Díaz-Rosales P., Romero A., Balseiro P., Dios S., Novoa B., Figueras A. (2012). Microarray-based identification of differentially expressed genes in families of turbot (*Scophthalmus maximus*) after infection with viral haemorrhagic septicaemia virus (VHSV). Mar. Biol.

[211] Cairns M.T., Johnson M.C., Talbot A.T., Pemmasani J.K., McNeill R.E., Houeix B., Sangrador-Vegas A., Pottinger T.G. (2008). A cDNA microarray assessment of gene expression in the liver of rainbow trout (*Oncorhynchus mykiss*) in response to a handling and confinement stressor. Comp. Biochem. Phys.

[212] Uchida M., Takumi S., Tachikawa K., Yamauchi R., Goto Y., Matsusaki H., Nakamura H., Kagami Y., Kusano T., Arizono K. (2012). Toxicity evaluation of glyphosate agrochemical components using Japanese medaka (*Oryzias latipes*) and DNA microarray gene expression analysis. J. Toxicol. Sci.

[213] Peatman E., Baoprasertkul P., Terhune J., Xu P., Nandi S., Kucuktas H., Li P., Wang S., Somridhivej B., Dunham R. (2007). Expression analysis of the acute phase response in channel catfish (*Ictalurus punctatus*) after infection with a Gram-negative bacterium. Dev. Comp. Immunol.

[214] Pridgeon J.W., Yeh H.Y., Shoemaker C.A., Mu X., Klesius P.H. (2012). Global gene expression in channel catfish after vaccination with an attenuated *Edwardsiella ictaluri*. Fish Shellfish Immunol.

[215] Pridgeon J.W., Yeh H.Y., Shoemaker C.A., Klesius P.H. (2012). Global transcription analysis of vaccinated channel catfish following challenge with virulent *Edwardsiella ictaluri*. Vet. Immunol. Immunop.

[216] Sun F., Peatman E., Li C., Liu S., Jiang Y., Zhou Z., Liu Z. (2012). Transcriptomic signatures of attachment, NF-κB suppression and IFN stimulation in the catfish gill following columnaris bacterial infection. Dev. Comp. Immunol.

[217] Collins J.E., White S., Searle S.M.J., Stemple D.L. (2012). Incorporating RNA-seq data into the zebrafish ensembl gene build. Genome. Res..

[218] Yang D., Liu Q., Yang M., Wu H., Wang Q., Xiao J., Zhang Y. (2012). RNA-seq liver transcriptome analysis reveals an activated MHC-I pathway and an inhibited MHC-II pathway at the early stage of vaccine immunization in zebrafish. BMC Genomics.

[219] Zhang Y., Stupka E., Henkel C.V., Jansen H.J., Spaink H.P., Verbeek F.J. (2011). Identification of common carp innate immune genes with whole-genome sequencing and RNA-seq data. J. Integr. Bioinforma.

[220] Sarropoulou E., Galindo-Villegas J., García-Alcázar A., Kasapidis P., Mulero V. (2012). Characterization of European sea bass transcripts by RNA seq after oral vaccine against *V. anguillarum*. Mar. Biotechnol.

[221] Salem M., Vallejo R.L., Leeds T.D., Palti Y., Liu S., Sabbagh A., Rexroad C.E., Yao J. (2012). RNA-seq identifies SNP markers for growth traits in rainbow trout. PLoS One.

[222] Li C., Zhang Y., Wang R., Lu J., Nandi S., Mohanty S., Terhune J., Liu Z., Peatman E. (2012). RNA-seq analysis of mucosal immune responses reveals signatures of intestinal barrier disruption and pathogen entry following *Edwardsiella ictaluri* infection in channel catfish, *Ictalurus punctatus*. Fish Shellfish Immunol.

[223] Evenhuis J., Bengten E., Snell C., Quiniou S.M., Miller N.W., Wilson M. (2007). Characterization of additional novel immune type receptors in channel catfish, *Ictalurus punctatus*. Immunogenetics.

[224] Montgomery B.C., Mewes J., Davidson C., Burshtyn D.N., Stafford J.L. (2009). Cell surface expression of channel catfish leukocyte immune-type receptors (IpLITRs) and recruitment of both Src homology 2 domain-containing protein tyrosine phosphatase (SHP)-1 and SHP-2. Dev. Comp. Immunol.

[225] Stafford J.L., Bengten E., du Pasquier L., Miller N.W., Wilson M. (2007). Channel catfish leukocyte immune-type receptors contain a putative MHC class I binding site. Immunogenetics.

[226] Liu H., Peatman E., Wang W., Abernathy J., Liu S., Kucuktas H., Lu J., Xu D.H., Klesius P., Waldbieser G. (2011). Molecular responses of calreticulin genes to iron overload and bacterial challenge in channel catfish (*Ictalurus punctatus*). Dev. Comp. Immunol.

[227] Liu H., Peatman E., Wang W., Abernathy J., Liu S., Kucuktas H., Terhune J., Xu D.H., Klesius P., Liu Z. (2011). Molecular responses of ceruloplasmin to *Edwardsiella ictaluri* infection and iron overload in channel catfish (*Ictalurus punctatus*). Fish Shellfish Immunol.

[228] Feng T., Zhang H., Liu H., Zhou Z., Niu D., Wong L., Kucuktas H., Liu X., Peatman E., Liu Z. (2011). Molecular characterization and expression analysis of the channel catfish cathepsin D genes. Fish Shellfish Immunol.

[229] Jiang Y., Abernathy J.W., Peatman E., Liu H., Wang S., Xu D.H., Kucuktas H., Klesius P., Liu Z. (2010). Identification and characterization of matrix metalloproteinase-13 sequence structure and expression during embryogenesis and infection in channel catfish (*Ictalurus punctatus*). Dev. Comp. Immunol.

[230] Sha Z., Xu P., Takano T., Liu H., Terhune J., Liu Z. (2008). The warm temperature acclimation protein Wap65 as an immune response gene: Its duplicates are differentially regulated by temperature and bacterial infections. Mol. Immunol.

[231] Huong Giang D.T., van Driessche E., Vandenberghe I., Devreese B., Beeckmans S. (2010). Isolation and characterization of SAP and CRP, two pentraxins from *Pangasianodon* (*Pangasius*) *hypophthalmus*. Fish Shellfish Immunol.

[232] Abernathy J., Xu D.H., Peatman E., Kucuktas H., Klesius P., Liu Z. (2011). Gene expression profiling of a fish parasite *Ichthyophthirius multifiliis*: Insights into development and senescence-associated avirulence. Comp. Biochem. Phys.

[233] Xu D.-H., Panangala V.S., van Santen V.L., Dybvig K., Abernathy J.W., Klesius P.H., Liu Z., Russo R. (2009). Molecular characteristics of an immobilization antigen gene of the fish-parasitic protozoanIchthyophthirius multifiliisstrain ARS-6. Aquac. Res.

[234] Lin A.F., Xiang L.X., Wang Q.L., Dong W.R., Gong Y.F., Shao J.Z. (2009). The DCSIGN of zebrafish: Insights into the existence of a CD209 homologue in a lower vertebrate and its involvement in adaptive immunity. J. Immunol.

[235] Lopez-Castejon G., Sepulcre M.P., Roca F.J., Castellana B., Planas J.V., Meseguer J., Mulero V. (2007). The type II interleukin-1 receptor (IL-1RII) of the bony fish gilthead seabream *Sparus aurata* is strongly induced after infection and tightly regulated at transcriptional and post-transcriptional levels. Mol. Immunol.

[236] Fan Y., Li S., Qi J., Zeng L., Zhong Q., Zhang Q. (2010). Cloning and characterization of type II interleukin-1 receptor cDNA from Japanese flounder (*Paralichthys olivaceus*). Comp. Biochem. Phys.

[237] Ilmiah Dana D., Pasaribu F.H., Affandi R. (2002). Increasing Thai catfish’s immunity (*Pangasius hypophthalmus* Fowler) using ascorbic acid. J. Akuakultur Indones.

[238] Xu D.-H., Klesius P.H., Peatman E., Liu Z. (2011). Susceptibility of channel catfish, blue catfish and channel × blue catfish hybrid to *Ichthyophthirius multifiliis*. Aquaculture.

[239] Mitchell A.J., Collins C. (1994). Enteric septicemia of catfish. Aquac. Mag.

[240] Watts M., Munday B.L., Burke C.M. (2001). Immune responses of teleost fish. Aust. Vet. J.

[241] Camp K.L., Wolters W.R., Rice C.D. (2000). Survivability and immune responses after challenge with *Edwardsiella ictaluri* in susceptible and resistant families of channel catfish, *Ictalurus punctatus*. Fish Shellfish Immunol.

